# Pharmacokinetic Characteristics of Nebulized Colistimethate Sodium Using Two Different Types of Nebulizers in Critically Ill Patients with Ventilator-Associated Respiratory Infections

**DOI:** 10.3390/antibiotics11111528

**Published:** 2022-11-01

**Authors:** Anna Kyriakoudi, Konstantinos Pontikis, Georgia Valsami, Stavrina Avgeropoulou, Efthymios Neroutsos, Eirini Christodoulou, Eleni Moraitou, Sophia L. Markantonis, Aristides Dokoumetzidis, Jordi Rello, Antonia Koutsoukou

**Affiliations:** 1 Intensive Care Unit, 1st Department of Pulmonology, Medical School, National & Kapodistrian University of Athens, General Hospital for the Diseases of the Chest “I Sotiria”, 11527 Athens, Greece; 2 Department of Pharmacy, School of Health Sciences, National & Kapodistrian University of Athens, 15784 Athens, Greece; 3 Microbiology Department, General Hospital for the Diseases of the Chest “I Sotiria”, 11527 Athens, Greece; 4 Clinical Research in Pneumonia (CRIPS), Vall d’Hebron Institute of Research, 08035 Barcelona, Spain; 5 Clinical Research, CHU Nîmes, 30900 Nîmes, France

**Keywords:** inhaled colistin, colistin pharmacokinetics, ELF colistin concentration, vibrating mesh nebulizer, jet nebulizer, critically ill

## Abstract

*Background:* Rising antimicrobial resistance has led to a revived interest in inhaled colistin treatment in the critically ill patient with ventilator-associated respiratory infection (VARI). Nebulization via vibrating mesh nebulizers (VMNs) is considered the current standard-of-care, yet the use of generic jet nebulizers (JNs) is more widespread. Few data exist on the intrapulmonary pharmacokinetics of colistin when administered through VMNs, while there is a complete paucity regarding the use of JNs. *Methods:* In this study, 18 VARI patients who received 2 million international units of inhaled colistimethate sodium (CMS) through a VMN were pharmacokinetically compared with six VARI patients who received the same drug dose through a JN, in the absence of systemic CMS administration. *Results:* Surprisingly, VMN and JN led to comparable formed colistin exposures in the epithelial lining fluid (ELF) (median (IQR) AUC_0–24_: 86.2 (46.0–185.9) mg/L∙h with VMN and 91.5 (78.1–110.3) mg/L∙h with JN). The maximum ELF concentration was 10.4 (4.7–22.6) mg/L and 7.4 (6.2–10.3) mg/L, respectively. *Conclusions:* Based on our results, JN might be considered a viable alternative to the theoretically superior VMN. Therapeutic drug monitoring in the ELF can be advised due to the observed low exposure, high variability, and appreciable systemic absorption.

## 1. Introduction

The increasing prevalence of multidrug resistance (MDR) among ventilator-associated Gram-negative infections, and the paucity of available therapeutic options, have led to suggestions of adjunctive strategies with inhaled antibiotics by scientific bodies such as the Infectious Diseases Society of America [1], while the European Society of Clinical Microbiology and Infectious Diseases holds a more restrained approach [2]. In any case, the matter remains highly controversial, especially upon publication of a number of studies that refute the efficacy of inhaled therapy in the setting of MDR infections affecting critically ill patients [3,4].

Polymyxins, namely, polymyxin B and polymyxin E (colistin), are cationic oligopeptide antimicrobials that were revived during the late 1990s and early 2000s for the treatment of Gram-negative infections in the face of rising carbapenem resistance [5,6]. No matter how pressing the need for them, they still possess significant toxicity—neurotoxicity, but especially nephrotoxicity, which according to several sources might affect a respected minority of patients, especially in the intensive care unit (ICU) setting.

In an effort to mitigate these toxicities, and building on the experience gathered in the field of cystic fibrosis, inhaled colistin treatment—in the form of nebulization of the inactive prodrug, colistimethate sodium (CMS)—emerged as a potential therapy for MDR and extensively drug resistant (XDR) infections [7]. The rationale has been the theoretic ability to achieve sufficient concentration in the infectious milieu of the lung parenchyma while sparing any significant systemic adverse outcome. In contrast to these theoretical considerations, inhaled CMS has only had modest efficacy and in secondary outcomes when tested in a randomized, controlled fashion [8,9]. However, in a meta-analysis of mostly observational data, the performance of adjunctive aerosolized colistin was associated with an approximately 60% increase and 40% decrease in the odds of clinical cure and infection-related mortality, respectively [10]. These discrepancies are reflected in official recommendations, with some guidelines advocating colistin aerosolization in ventilator-associated pneumonia (VAP) as an adjunct to (and not instead of) intravenous therapy [1,11], while some others do not [2].

Currently, inhaled colistin (either in the form of CMS or colistin sulphate) is the most frequently used nebulized antimicrobial treatment in ICUs globally; it is estimated that more than two-thirds of ICUs are actively using this treatment [12]. This is in sharp contrast with the evidence on its efficacy, and lack thereof, and the insufficient knowledge of the pharmacokinetics of CMS/colistin in the lung. Indeed, only a handful of studies have been published that deal with the pharmacokinetics of colistin in the respiratory system, altogether comprising 64 patients [13,14,15,16]. Furthermore, published experience only refers to nebulization via vibrating mesh nebulizers (VMNs) with a complete paucity of data on jet nebulization. In the literature, the superiority of VMNs over jet nebulizers (JNs) is supported by in vivo and in vitro studies, indicating advantages of the former consisting of a higher respirable inhaled mass [17], a greater deposition to the lung parenchyma, and a lower residual volume that allows the administration of higher doses in a smaller amount of time [18]. On the other hand, VMNs are not widely available, especially in resource-limited environments, and JNs remain the most frequently used modality in ICUs worldwide [19]. As such, any information on the comparative pharmacokinetic (PK) features of these technologies is of undisputable value and forms the objective of this study.

## 2. Results

**Figure 1:**
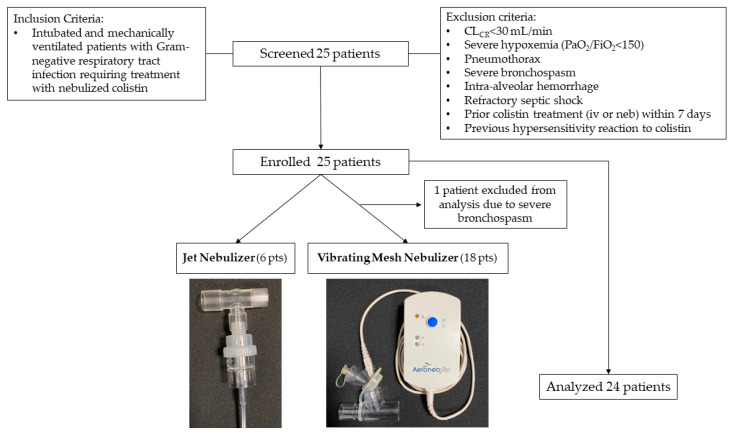
Patient flow chart. iv: intravenous, neb: nebulized.

Twenty-five patients were consented for study participation. One patient developed severe bronchospasm upon CMS inhalation completion, which precluded the performance of the lavage procedure. Thus, a total of 24 patients with ventilator-associated tracheobronchitis (VAT, *n* = 10) or VAP (*n* = 14) were enrolled in the study. Six patients received CMS via JN and 18 via VMN. The patient flow chart is depicted in Figure 1. The characteristics of the study population are shown in Table 1. Patients in the VMN group were older, with higher admission Acute Physiology and Chronic Health Evaluation II (APACHE II) scores, however, had a higher PaO_2_/FiO_2_ ratio on study entry. Ventilator-associated pneumonia represented 61% and 50% of the diagnosed infections in the VMN and JN groups, respectively.

**Figure 2:**
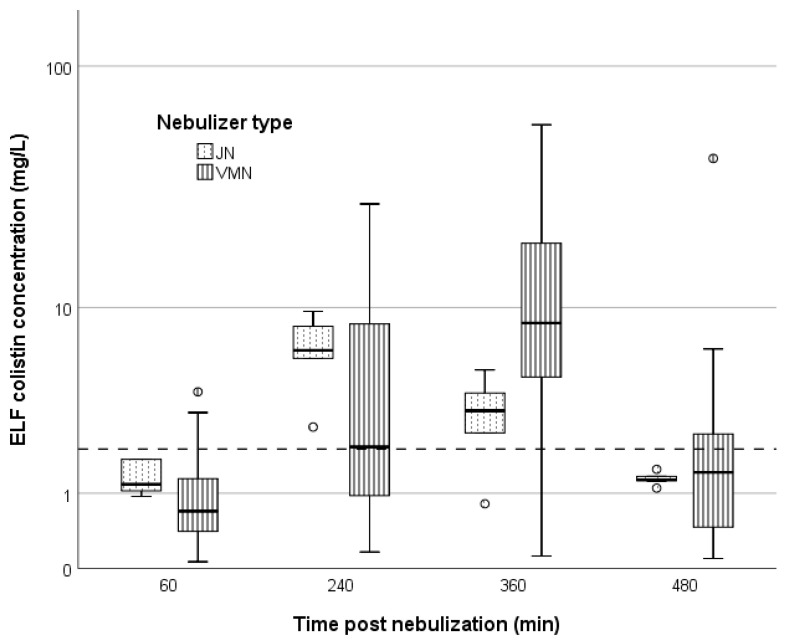
Colistin concentration in the ELF at 60, 240, 360 and 480 min after inhalation of 2 million international units of colistimethate sodium via jet nebulizers (dotted boxplots) or vibrating mesh nebulizers (vertical line boxplots). The dashed line represents the European Committee on Antimicrobial Susceptibility Testing (EUCAST) breakpoint for susceptible *Enterobacterales* and *Acinetobacter* spp. (2 mg/L). Circles represent outliers. ELF: epithelial lining fluid, JN: jet nebulizers, VMN: vibrating mesh nebulizers.

The achieved colistin concentration in the epithelial lining fluid (ELF) (C_colistin,ELF_) at consecutive time points with the two modalities under comparison are shown in Figure 2. Median (IQR) values of C_colistin, ELF_ obtained with VMN were 0.7 (0.4–1.3) mg/L, 2.1 (1.0–8.5) mg/L, 8.6 (4.8–18.9) mg/L, and 1.42 (0.5–2.5) mg/L, at 1, 4, 6 and 8 h post-nebulization, respectively. Median values of C_colistin, ELF_ achieved with JN at the same time points were 1.2 (1.0–1.7) mg/L, 6.42 (5.9–8.3) mg/L, 3.3 (2.5–4.0) mg/L and 1.3 (1.2–1.3) mg/L, respectively.

As shown in Figure 2, both nebulizers achieved concentrations in the ELF higher than the European Committee on Antimicrobial Susceptibility Testing (EUCAST) minimum inhibitory concentration (MIC) breakpoint for *Enterobacterales* and *Acinetobacter* spp. [20], with the effect being more pronounced for the VMN device and the 4 h and 6 h timepoints.

**Figure 3:**
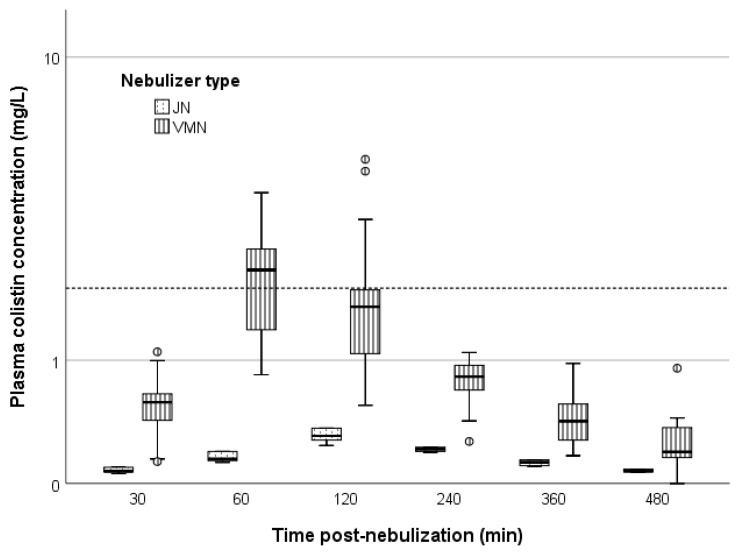
Colistin plasma concentration at 30, 60, 120, 240, 360 and 480 min after inhalation of 2 million international units of colistimethate sodium via jet nebulizers (dotted boxplot) and vibrating mesh nebulizers (vertical line boxplot). The dashed line represents the average steady-state plasma colistin concentration (2 mg/L) associated with the occurrence of acute renal failure according to [21]. Circles represent outliers. JN: jet nebulizers, VMN: vibrating mesh nebulizers.

The median (IQR) values of colistin concentration in plasma (C_colistin, plasma_) using VMN nebulization were 0.6 (0.4–0.7) mg/L, 2.3 (1.4–2.7) mg/L, 1.7 (1.1–2.0) mg/L, 0.8 (0.7–0.9) mg/L, 0.4 (0.3–0.6) mg/L, and 0.2 (0.2–0.4) mg/L, at 0.5, 1, 2, 4, 6 and 8 h post-nebulization. The corresponding values with JN were 0.1 (0.1–0.1) mg/L, 0.1 (0.1–0.2) mg/L, 0.3 (0.3–0.4) mg/L, 0.2 (0.2–0.2) mg/L, 0.1 (0.1–0.1) mg/L, and 0.1 (0.1–0.1) mg/L (Figure 3). Median C_colistin, plasma_ was greater than 2 mg/L at 1 h post-nebulization, a value which is the average concentration, in steady-state conditions, that predicts acute renal failure [21,22]. Such an occurrence was not evident for nebulization via the JN device (Figure 3).

Pharmacokinetic parameters of colistin in the ELF and plasma are shown in Table 2. Median maximum concentration (C_max_) in the ELF was arithmetically greater in the VMN group than in the JN group (10.4 vs 7.4 mg/L, respectively), however the difference was not statistically significant (*p*: 0.549). A later peak (by approximately 2 h) was observed with the VMN device. Accordingly, while at 2 h from the index nebulization, a greater percentage of JN patients had ELF concentrations over 2 mg/L (the EUCAST breakpoint for colistin susceptibility among *Acinetobacter* spp. and *Enterobacterales*), this was reversed at 6 and 8 h. Considering the EUCAST breakpoint for *Pseudomonas* spp. (4 mg/L), JN-treated patients had more frequently at 4 h, and less frequently at 6 h post-nebulization, exceeding ELF concentrations. Overall, the ELF exposure achieved with the two devices was comparable, and their difference was not statistically significant (*p*: 0.871).

Regarding the plasma compartment, a greater amount of formed colistin was observed in the VMN group, as evidenced by both the higher area under the curve (AUC) and C_max_. At various timepoints, a considerable percentage of patients had plasma concentrations over 2 mg/L, a value associated with acute renal failure [21,22], with the effect being almost exclusively present in the VMN group. The ELF/plasma mean concentration ratio was comparable between the two groups (5.5 vs 4.8 for VMN and JN, respectively).

### Safety

There were no instances of clinically important deterioration of oxygenation during the nebulization. One patient developed bronchospasm upon completion of CMS inhalation via VMN and was treated promptly by inhaled β2-agonists and ipratroprium, which led to a rapid recovery.

## 3. Discussion

In this single-center study, we compared the pharmacokinetic properties of formed colistin in the ELF and the plasma compartment when inhaled CMS, at a typical dose, was administered either via a VMN or a generic JN. According to our results, the two devices were associated with comparable exposures in the lung, of a level, however, that will be doubtful in leading to desirable clinical results. Indeed, the median total (as opposed to unbound) AUC_0–24_ in the ELF for the two modalities were 86.2 and 91.5 mg/L*h for VMN and JN, respectively. Assuming an unbound fraction of colistin in the ELF of 0.05 to 0.1 [16], this translates to a free colistin exposure that would be 10 to 20-fold lower and in the range of 5–10 mg/L∙h for both devices. Relatively recent evidence suggests that the optimal pharmacokinetic/pharmacodynamic index for colistin is the area under the unbound colistin concentration curve divided by the pathogen MIC (fAUC/MIC) [23,24]. At typical plasma maximum concentrations, an fAUC/MIC of 25 has been linked to 2-log killing in in vitro experiments for *Klebsiella pneumoniae* [23], while values of 12.2–22.8 and 7–42 have been found to predict 1-log killing in *Pseudomonas aeruginosa* and *Acinetobacter baumannii* animal thigh and lung infection models [24]. Assuming that these considerations and hypotheses are true, and given the rising MICs to colistin among critically ill patients in endemic environments [25] (where this inhalational antimicrobial treatment is needed the most), it is highly unlikely that for the “median” patient the AUCs measured herein will have any positive impact on the infection outcome. Indeed, suboptimal exposure might lie behind reported failures of inhaled polymyxins to objectively improve clinical outcomes [8,26].

The equivalence of exposure of the two devices came as a surprise, since evidence has suggested that VMNs are linked to higher exposures for several reasons [27]. The number of patients that received JN-delivered treatment was small, however these results provide a basis for JN nebulization of CMS in areas where VMNs are not available.

JN nebulization led to an earlier peak of ELF concentration, a similar exposure and a slightly lower C_max_. Indeed, at 4 h post-nebulization, six out of six JN-treated patients had ELF concentrations over 2 mg/L, while five out of six had values over 4 mg/L. The speed of peak concentration attainment might be relevant in critical settings. If these findings are verified in a larger population, then higher doses and shorter between-dose intervals in JN-treated patients (as opposed to VMN-treated) might be worth considering.

Our results were in concordance with a previous study in a similar ICU setting, where administration of 1 mIU of CMS via VMN led to a median (IQR) C_max_ of 6.7 (4.8–10.1) mg/L [13], while inhalation of the double dose in our study led to a median C_max_ of approximately 1.5-fold. The same pattern was evident in systemic absorption and plasma levels (median C_max_ was 2.6 mg/L in our study vs. 1.6 mg/L in the Athanassa et al. paper [13]). However, the ELF exposure was similar, despite the higher dose, probably due to a higher clearance from the lung compartment in our study, as evidenced by the lower C_min_. The reason for this discrepancy is not straightforward; although, it might be due to the inter-patient variability of CMS/colistin pharmacokinetics, disease characteristics, the mode of the mechanical ventilation application, or differences in the manufacturing process of CMS over the years.

Contrarily, our results were only marginally compatible with the results of Boisson et al. [14] and Gkoufa et al. [16]. In the former study, the range of colistin concentration in the ELF following a 2 mIU inhalation spanned from 9.53 to 1137 mg/L [14], while it was between 0.8 mg/L and 57.9 mg/L in the present study. In the latter study, the exact values were not reported, however in the figured data it was shown that after an inhalation of 3 to 5 mIU of CMS, formed colistin concentrations were in the range of 10^2^ to 10^3^ mg/L [16]. Again, it is not known whether these differences were due to pre-analytical/analytical procedures or were the result of variability of colistin pharmacokinetics, which has been observed in repeated instances [14,16]. Furthermore, despite the higher colistin ELF concentration in these studies, the systemic absorption was far less compared with our observations. Indeed, the plasma concentrations in the two papers were generally below 1 mg/L [14,16], whereas in our study 50% of the VMN-treated population had plasma C_max_ over 2.6 mg/L, and 25% had values over 3.5 mg/L. In any case, if such values are substantiated in future research, then this should alert clinicians to the danger of acute renal failure in nebulized CMS-treated patients, since formed colistin plasma values over 2–2.5 mg/L have been linked to the occurrence of nephrotoxicity [21,22].

Nevertheless, in a piglet model of ventilated *P. aeruginosa* inoculation pneumonia, Lu et al. observed a serum maximum colistin concentration of 1.6 ± 1.4 mg/L after VMN aerosolization of 0.1 mIU/kg of CMS [28]. Upon animal sacrifice, 1 h after the third inhaled dose, it was noted that the median peak lung tissue concentration was 2.8 μg/g, suggesting that a certain level of systemic absorption is required for a sufficient colistin amount to reach the extracellular space of lung parenchyma [28]. Post-nebulization colistin tissue levels have never been measured in humans; if the results above are reproducible in the human host, and given that Lu et al. observed a positive infection outcome in their animal model with inhaled treatment, one should acknowledge not only the risks of systemic absorption, but also the benefit of it as a side effect of desirable tissue penetration.

The inter-patient variability in colistin ELF exposure that we and others have observed [14,15,16], and the importance of the exposure to the infection outcome [23], probably mandates that every effort should be made for therapeutic drug monitoring (TDM) to be applied in difficult-to-treat infections with demanding pathogens. It is worth noting that this variability was more pronounced in VMN-treated patients. With a similar rationale, TDM has been advocated for systemic CMS administration [11,23,29], and given the safety and costs of the mini-bronchoalveolar lavage (BAL) procedure, “respiratory TDM” for colistin should be considered as an option.

The strengths of our study are the strict clinical, pre-analytical and analytical protocol that vouch for the internal validity of our findings. Furthermore, patients were naïve of systemic CMS administration, so confounding by recent or remote receipt of the drug was totally avoided. One limitation of our research was the relatively smaller size of the JN population, owing to the pragmatic design of the study. Additional weaknesses were the decision to forfeit collection of outcome data and the imbalances between the two groups. An example of the latter was the non-statistically significant (*p*: 0.177) 12 year difference in median age between the two groups. However, the modest expected decline in pulmonary function in such a duration [30] and the strict nebulization process in patients that were mechanically ventilated argue against a significant effect on our results. All these shortcomings were partially justified by logistical issues and by our specific aim of comparing the pharmacokinetics associated with the two devices. Additionally, we opted for using mini-BAL to approach lung pharmacokinetics. It is well-known that the lavage might be contaminated by tracheal secretions [31,32], however until microdialysis techniques become widely available [33], bronchoalveolar lavage is the only, logistically feasible, option. Nevertheless, the results presented herein should ideally be verified by in vivo microdialysis studies. Furthermore, we did not measure CMS, which could have helped us elucidate the differences with other studies that we noted [14,16]; and, lastly, we did not build a population pharmacokinetic model.

## 4. Materials and Methods

### 4.1. Study Population

The study population consisted of 24 critically ill adult patients, admitted to the intensive care unit of the First Department of Pulmonology of the National and Kapodistrian University of Athens between June 2014 and November 2017. All patients were intubated and mechanically ventilated, and received inhaled CMS as part of their treatment for VAP or VAT caused by Gram-negative pathogens. Subjects were studied around the first dose of nebulized treatment, which always preceded intravenous treatment, to avoid confounding by colistin (or CMS) reaching the ELF via the systemic circulation. We excluded patients that received a dose different to the institution’s standard of 2 million international units (mIU) (~160 mg) of CMS and patients who were planned to receive intravenous colistin within 8 h of nebulization initiation. We also excluded patients with: (i) a creatinine clearance (CR_CL_) of less than 30 mL/min, (ii) severe hypoxemia defined as PaO_2_/FiO_2_ of less than 150, (iii) pneumothorax, (iv) severe bronchospasm, (v) intra-alveolar hemorrhage, (vi) refractory septic shock, (vii) prior colistin treatment, either intravenously or by inhalation, within 7 days prior to index nebulization and, (viii) previous hypersensitivity reaction to colistin (Figure 1).

Demographic, clinical, and microbiological characteristics of the study population were recorded on the first day of nebulized CMS administration.

### 4.2. Study Procedures

A dose of 2 mIU of CMS (Colistin^®^, Norma Hellas, Athens, Greece), corresponding to approximately 160 mg CMS or 68 mg colistin-base activity, was nebulized over a 10–30 min period via either VMN (Aeroneb Solo with Aeroneb Pro controller, Aerogen, Galway, Ireland) or generic JN. This was not a randomized trial, and the nebulizer selection was exclusively a matter of availability of VMN consumables in the ICU at the time. The intended dose was dissolved in 4 mL of normal saline 0.9%. The nebulizer was placed at the inspiratory limb of the ventilator circuit, at 15 cm from the Y-piece, and the heat and moisture exchanger was removed. During nebulization, the patients were sedated and ventilated using assist volume controlled mode, with tidal volume set at 8 mL/kg of ideal body weight, a constant squared-wave inspiratory flow, a positive end expiratory pressure (PEEP) of less than or equal to 10 cm H_2_O, an inspiratory to expiratory time ratio equal to 1, an inspiratory pause of 10%, and a respiratory rate set at 15/min [31].

Mini BAL was performed just before, and at 1, 4, and 8 h after the index nebulization, via a 65 cm long, 12 F diameter sterile catheter (Kimberly-Clark, Roswell, GA, USA). The catheter was inserted through the endotracheal tube. The lavage was performed with instillation of 2 syringes of 20 mL sterile normal saline at room temperature through the catheter. The aspirate from the first syringe was considered representative of bronchial washing and was, therefore, discarded. The aspirate from the second syringe was collected and centrifuged in frigid centrifuge (3500× *g* for 10 min at 4 °C) immediately (<2 min) to avoid falsely elevated levels of urea in BAL [34] and CMS hydrolysis to formed colistin. The supernatant liquid was stored in plastic micro test tubes (Safe-lock; Eppendorf AG; Hamburg, Germany) and frozen at –80 °C, until analysis.

Blood samples (5 mL) were collected through a peripheral arterial line just before and at 0.5, 1, 2, 4, and 8 h post-nebulization initiation. The specimens were immediately centrifuged (3500× *g* for 10 min) in a frigid centrifuge (at 4 °C) and supernatants were collected in Eppendorfs and were frozen at −80 °C until analysis.

### 4.3. Colistin Measurement Assay in Plasma and BAL Fluid

Colistin concentrations in plasma (C_colistin,plasma_) and epithelial lining fluid (C_colistin,ELF_) were measured through high performance liquid chromatography (HPLC)-fluorescence analysis.

#### 4.3.1. Instrumentation

The liquid chromatographic system consisted of a Shimadzu LC-20 AD pump with a DGU-20 A_5R_ degasser, a SIL-HT thermostated auto sampler, a CTO-20 AC oven and an RF-20 A prominence fluorescence detector.

#### 4.3.2. Plasma and BAL Fluid Assay

The samples were rapidly centrifuged, and plasma and BAL supernatant were separated and kept frozen (−80 °C) before analysis, as previously described. An HPLC-RF assay was used for colistin concentration measurements in plasma and BAL fluid samples, based on the method of Li et al. [35], as modified by Markou et al. [36] and Ratjen et al. [37]. Briefly, netilmicin was used as the internal standard and the method consisted of a protein precipitation step with trifluoracetic acid (TFA), followed by colistin derivatization with 9-fluorenylmethyl chloroformate on a solid phase extraction C18 cartridge. The eluted fluid was dried under vacuum and the solid residue was reconstituted in mobile phase for subsequent reversed-phase HPLC with fluorescence detection (excitation: 260 nm; emission: 316 nm). The analytical column was a Phenomenex Luna C18, 3 μm, 50 mm × 3 mm with 100 Å particle size, while the mobile phase consisted of ACN/THF/H_2_O (80/7.7/12.3%) with a flow rate of 0.5 mL/min. The injection volume was 20 μL. The method was linear at a concentration range of 0.10–4.8 mg/L (R^2^ = 0.999). The limit of detection (LOD) and lower limit of quantification (LLOQ) were 0.01 and 0.04 mg/L, respectively, while the intra- and inter-assay variabilities were less than 16%. Representative chromatograms and calibration curves in plasma and BAL are given in the Supplementary Material (Figures S1 and S2).

#### 4.3.3. Urea Plasma and BAL Assay

Urea concentration in plasma (Urea _plasma_) and BAL (Urea _BAL_) of all patients were also measured, applying an enzymatic UV method, and the ratio of Urea _plasma_/Urea _BAL_ was used as a dilution factor to correct measured colistin concentration in BAL and calculate actual ELF concentrations as:

(1) $$C_{\mathrm{ELF}}=C_{\mathrm{BAL}}\times \frac{{\mathrm{Urea}}_{\;\mathrm{plasma}}}{{\mathrm{Urea}\;}_{\mathrm{BAL}}}$$

#### 4.3.4. PK Analysis

PK analysis was performed on measured serum and ELF concentrations using the Phoenix^®^ version 8.3 PK/PD software package (Certara, Princeton, NJ, USA).

Noncompartmental analysis (NCA) was applied to plasma and ELF colistin concentration-time curves and basic PK parameters were calculated, namely, the maximum observed plasma and ELF colistin concentration (C_max_) and the time C_max_ was observed (T_max_) for each patient. The area under the concentration–time curves from time zero to the last experimental point (AUC_0–t_) was obtained by the log-linear trapezoidal method. The last observed concentration divided by the terminal slope λ (k_el_), was used to calculate the area under the concentration–time curve extrapolated to infinity (AUC_inf_). The terminal slope was estimated by linear regression analysis on the last three points of the log-transformed concentrations vs. time plot. The elimination half-life was calculated as t_1/2_ = 0.693/λ. Total apparent clearance (CL/F) and volume of distribution (V_d_/F) were evaluated as dose/AUC_inf_, and dose/(λ × AUC_inf_), respectively. AUC_0–24_ was calculated as AUC_inf_ × 3 (for thrice daily administration).

### 4.4. Safety

Subjects were medically followed during the nebulization phase and the post-nebulization period for the development of side effects, especially those related to the respiratory system. In cases of significant hypoxemia and/or bronchospasm, the nebulization and lavage processes were suspended, and the patient was treated as clinically indicated.

### 4.5. Statistical Analysis

Statistical analysis was performed using IBM’s SPSS statistics v.24.0. Data were summarized as frequencies or as the mean value ± standard deviation (SD), or as the median and interquartile range (IQR), as appropriate. A Mann–Whitney non-parametric U-test was performed to compare differences between groups, and Fisher’s exact test was employed for the comparisons between frequencies. A p-value of 0.05 was considered as the threshold of statistical significance.

### 4.6. Protocol Approval and Ethical Considerations

The clinical protocol was reviewed and approved by the Institutional Review Board (IRB) of the General Hospital for the Diseases of the Chest “I Sotiria” (Approval numbers: 6764/15.03.2013, 04155/26.05.2015 and 1132/18.01.2017—original protocol and subsequent amendments). The IRB consists of nine health professionals (physicians and nurses) with discrete levels of hierarchy, and is imbued with the role of an ethics committee. Approval was also provided by the Special Research Account of the National and Kapodistrian University of Athens (ELKE, Approval Number 12483) which was also responsible for the financial management of the study, in accordance with local regulations. All patients were mechanically ventilated and unconscious and, thus, written informed consent was obtained from their legally acceptable representatives, as per local legislation. The study was sponsored by Norma Hellas, however the conduct of the study, the decision to submit the manuscript and the content of this report were exclusively a responsibility of the investigators.

This work was presented, in part, in the American Thoracic Society International Congress held in Philadelphia, TN, USA. between 15 and 20 May 2020 [38].

## 5. Conclusions

Given the assumptions and limitations of the current study, inhalation of 2 mIU of CMS led to similar exposures when administered either via VMN or JN nebulization. The latter resulted in lower colistin plasma levels, below the average steady-state plasma colistin concentration (2 mg/L) associated with the occurrence of acute renal failure. Accordingly, JN appears as a viable substitute of VMN in cases of availability issues, however a different dose and interval might be considered. Apart from the comparison between devices, the measured ELF exposure casts doubt on whether the selected dose might be relied upon for difficult-to-treat infections, while, contrary to most published evidence, systemic colistin absorption was not negligible, especially with the vibrating mesh technology.

## Figures and Tables

**Table 1:** Patient baseline characteristics. VMN: vibrating mesh nebulizer; JN: jet nebulizer; IQR: interquartile range; APACHE II: Acute Physiology and Chronic Health Evaluation II score; VAP: ventilator-associated pneumonia; VAT: ventilator-associated tracheobronchitis.

Characteristic		VMN (*n* = 18)	JN *(n = 6)*
Median age in years (IQR)		69.5 (56.8–83.0)	57.5 (55.5–67.3)
Female gender, *n* (%)		5 (27.8)	1 (16.7)
Caucasian race, *n* (%)		18 (100)	6 (100)
Mean admission APACHE II (SD)		21.4 (6.3)	18.2 (6.0)
Type of infection (%)	VAP	11 (61.1%)	3 (50%)
	VAT	7 (38.9%)	3 (50%)
Isolated pathogen (%)	*A.baumannii*	11 (61.1%)	3 (50%)
	*K.pneumoniae*	2 (11.1%)	
	Both	2 (11.1%)	
	Culture negative	3 (16.7%)	3 (50%)
Median creatinine clearance, mL/min (IQR)		53.0 (40–65.5)	86 (67.5–122.0)
Mean PaO_2_/FiO_2_ (SD)		258.7 (88.0)	190.3 (68.1)

**Table 2:** Pharmacokinetic/pharmacodynamic (PK/PD) parameters in epithelial lining fluid (ELF) and in plasma after inhalation of 2 million international units of colistimethate sodium via jet nebulizer (JN) or vibrating mesh nebulizer (VMN). Values are expressed as frequencies or medians and interquartile ranges, unless otherwise specified. Comparisons with statistically significant differences are depicted in bold.

PK/PD parameter	ELF			Plasma		
	VMN	JN	*p* Value	VMN	JN	*p* Value
AUC_0–8 h_, mg/L∙h	28.7 (15.1–68.1)	30.5 (26.0–36.8)	0.871	**7.5 (5.5–8.8)**	**1.3 (1.3–6.4)**	**0.039**
AUC_0–24_, mg/L∙h	86.2 (46.0–185.9)	91.5 (78.1–110.3)	0.871	**22.45 (±9.1)**	**4.0 (3.8–19.3)**	**0.039**
C_colistin_ over 2 mg/L						
30 min, %				0	0	
60 min, %	16.7	16.7	1.0	61.1	16.7	0.155
120 min, %				22.2	16.7	1.0
240 min, %	50	100	0.052	5.6	16.7	0.446
360 min, %	88.9	83.3	1.0	0	0	
480 min, %	41.2	0	0.124	0	0	
C_colistin_ over 4 mg/L						
60 min, %	5.6	16.7	0.446			
240 min, %	**27.8**	**83.3**	**0.050**			
360 min, %	**83.3**	**33.3**	**0.038**			
480 min, %	17.6	0	0.539			
C_max_, mg/L	10.4 (4.7–22.6)	7.4 (6.2–10.3)	0.549	**2.6 (2.0–3.5)**	**0.3 (0.3–1.6)**	**0.016**
C_min_, mg/L	**0.4 (0.1–0.8)**	**1.1 (0.9–1.2)**	**0.006**	**0.2 (0.1–0.3)**	**0.1 (0.1–0.2)**	**0.016**
T_max_, h	**6.5 (6.3–6.6)**	**4.3 (3.5–4.3)**	**0.003**	**1.5 (1.4–1.7)**	**2.2 (2.1–2.2)**	**0.013**
Volume of distribution/ fraction of dose absorbed (L)	1.7 (0.8–5.2)	4.5 (3.5–5.1)	0.141	**22.8 (16.8–33.0)**	**129.8 (95.6–151.9)**	**0.016**
Clearance/fraction of dose absorbed (L/h)	1.4 (0.8–3.6)	1.8 (1.5–2.1)	0.779	**7.3 (6.0–9.1)**	**37.6 (23.2–39.9)**	**0.016**
Mean T_1/2_, h (SD)	-	-		2.4 (1.1)	2.4 (0.5)	
Mean ELF/Plasma ratio (SD)	5.5 (13.2)	4.8 (1.4)				

## Data Availability

Not applicable.

## Supplementary Materials

The following are available online at https://www.mdpi.com/article/10.3390/antibiotics11111528/s1: Figure S1. Representative chromatograms of Colistin and Netilmicin (I.S.) in plasma (A) and BAL (B). Figure S2. Representative calibration curves in plasma (A) and BAL (B).
