# Supplementary material for: Pharmacokinetic Characteristics of Nebulized Colistimethate Sodium Using Two Different Types of Nebulizers in Critically Ill Patients with Ventilator-Associated Respiratory Infections
Source: Antibiotics (Basel). 2022 Nov 1;11(11):1528. doi: 10.3390/antibiotics11111528 (PMC9686516; doi:10.3390/antibiotics11111528)
Supplement: Supplementary file 1 [file antibiotics-11-01528-s001.zip › antibiotics-1974970-supplementary.pdf]

Supplementary material

Pharmacokinetic characteristics of nebulized Colistimethate Sodium using two different types of nebulizers in critically ill patients with ventilator-associated respiratory infections

Anna Kyriakoudi [1,†] Konstantinos Pontikis [1,*,†], Georgia Valsami [2], Stavrina Avgeropoulou [1], Efthymios Neroutsos [2], Eirini Christodoulou [2], Eleni Moraitou [3], Sophia L. Markantonis [2], Aristides Dokoumetzidis [2], Jordi Rello [4] and Antonia Koutsoukou [2]

[1] Intensive Care Unit, 1 st Department of Pulmonology, Medical School, National & Kapodistrian University of Athens, General Hospital for the Diseases of the Chest "I Sotiria", 11527 Athens, Greece
[2] Department of Pharmacy, School of Health Sciences, National & Kapodistrian University of Athens, 15784 Athens, Greece
[3] Microbiology Department, General Hospital for the Diseases of the Chest "I Sotiria", 11527 Athens, Greece
[4] Clinical Research in Pneumonia (CRIPS), Vall d'Hebron Institute of Research, 08035 Barcelona, Spain
[5] Clinical Research, CHU Nîmes, 30900 Nîmes, France
* Correspondence: pontikis.k@sotiria.gr
† These authors contributed equally to this work.

Nebulizer types

Throughout this study vibrating mesh nebulization was performed with an Aeroneb Solo nebulizer and an Aeroneb Pro Controller (Aerogen, Galway, Ireland). The serial number of the controller was AP-1403486. The lot number of the Aeroneb Solo nebulizers was: LOT 9931140513036. Generic jet nebulizers connected to RTM3 France flow meters were used for jet nebulization.

Representative chromatograms and calibration curves

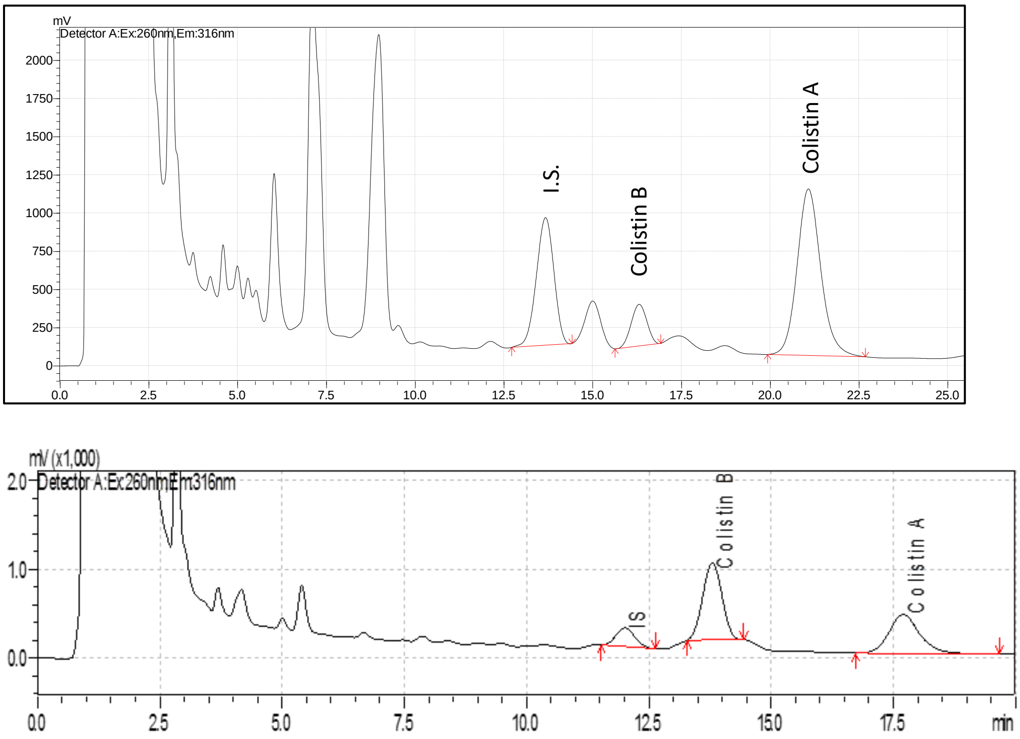

A

B

Figure S1. Representative chromatograms of Colistin and Netilmicin (I.S.) in plasma (**A**) and BAL (**B**)

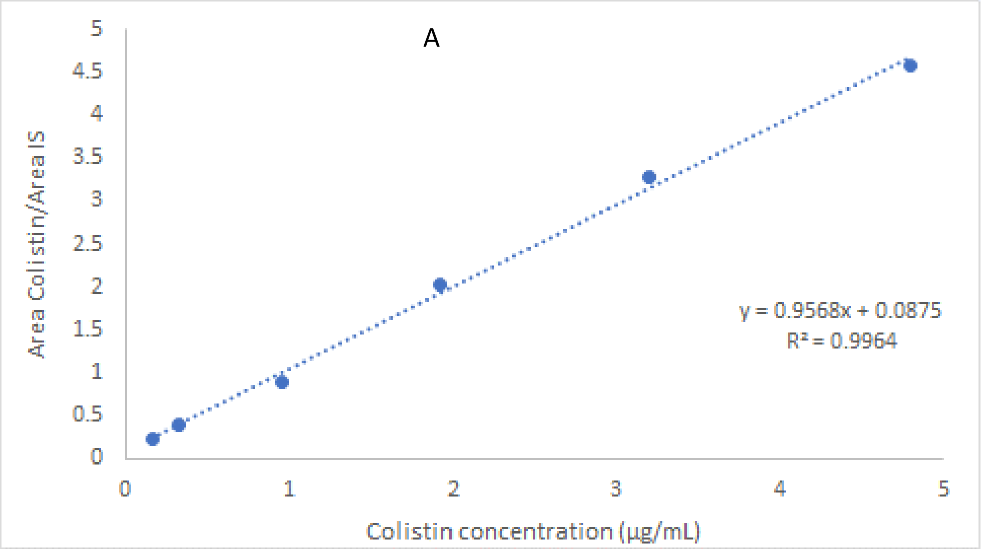

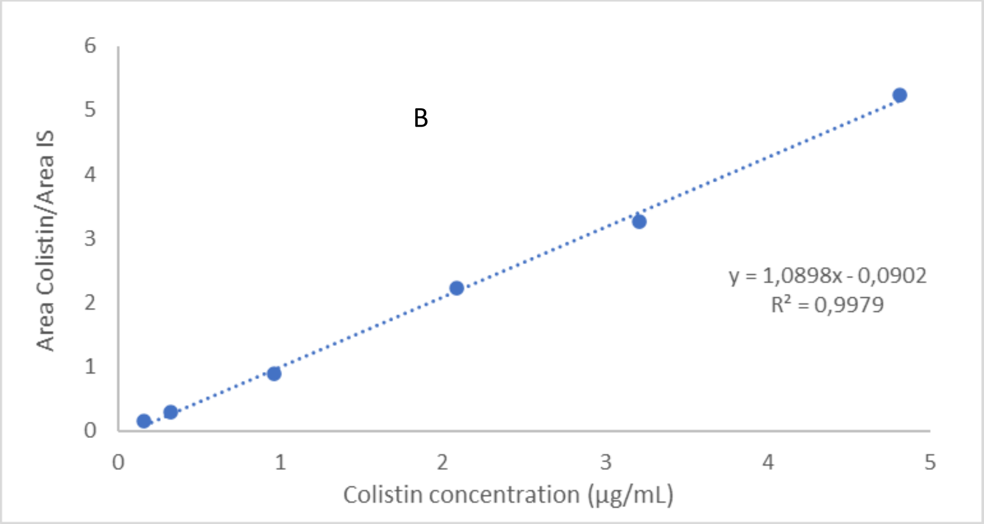

Figure S2. Representative calibration curves in plasma (**A**) and BAL (**B**).
